# Clonality disguises the vulnerability of a threatened arid zone Acacia

**DOI:** 10.1002/ece3.3246

**Published:** 2017-10-11

**Authors:** David G. Roberts, Cairo N. Forrest, Andrew J. Denham, David J. Ayre

**Affiliations:** ^1^ School of Biological Sciences and Centre for Sustainable Ecosystem Services University of Wollongong Wollongong NSW Australia; ^2^ New South Wales Office of Environment and Heritage Hurstville NSW Australia; ^3^ Present address: Centre of Excellence in Natural Resource Management The University of Western Australia Albany WA Australia

**Keywords:** Australia, clonal plants, conservation, heterozygosity, polyploidy, population genetics, reproductive failure

## Abstract

Long‐lived, widespread plant species are expected to be genetically diverse, reflecting the interaction between large population sizes, overlapping generations, and gene flow. Such species are thought to be resilient to disturbance, but may carry an extinction debt due to reproductive failure. Genetic studies of Australian arid zone plant species suggest an unusually high frequency of asexuality, polyploidy, or both. A preliminary AFLP genetic study implied that the naturally fragmented arid zone tree, *Acacia carneorum*, is almost entirely dependent on asexual reproduction through suckering, and stands may have lacked genetic diversity and interconnection even prior to the onset of European pastoralism. Here we surveyed microsatellite genetic variation in 20 stands to test for variation in life histories and further assessed the conservation status of the species by comparing genetic diversity within protected stands in National Parks and disturbed range lands. Using herbarium records, we estimate that 219 stands are extant, all of which occur in the arid zone, west of the Darling River in southeastern Australia. With two exceptions, all surveyed stands comprised only one multilocus genet and at least eight were putatively polyploid. Although some stands comprise thousands of stems, our findings imply that the species as a whole may represent ~240 distinct genetic individuals, many of which are polyploid, and most are separated by >10 km of unsuitable habitat. With only 34% of stands (and therefore genets) occurring within conservation reserves, *A. carneorum* may be at much greater risk of extinction than inferred from on‐ground census data. Land managers should prioritize on‐ground preservation of the genotypes within existing reserves, protecting both vegetative suckers and seedlings from herbivory. Importantly, three stands are known to set viable seed and should be used to generate genetically diverse germ‐plasm for ex situ conservation, population augmentation, or translocation.

## 1 INTRODUCTION

Remnant stands of long‐lived, recently abundant, and widespread plant species, in particular, temperate, and tropical forest trees, are often found to display high levels of genetic diversity, reflecting genetic interconnection, mediated by pollen or seed dispersal (Kramer, Ison, Ashley, & Howe, 2008) within typically large populations. However, such species may carry an extinction debt (Janzen, 1986) from limited or complete reproductive failure, for example, due to compatible mating‐partner limitation in now small or isolated stands. Canopy‐forming trees, shrubs and some animal species in arid habitats, including those that are locally abundant or common, are often found to exhibit asexuality, polyploidy, or both. Examples include the creosote bush of South West USA and northern Mexico (Vasek, 1980) and rock cresses of the northern USA (Mau et al., 2015), Saharan olives (Besnard & Baali‐Cherif, 2009), and some Australian arid zone plant and animal species, including acacias (Andrew, Miller, Peakall, Crisp, & Bayer, 2003; Roberts et al. 2016), mallee eucalypts (Bradbury, Grayling, MacDonald, Hankinson, & Byrne, 2016; Kennington & James, 1997; Rossetto, Jezierski, Hopper, & Dixon, 1999), *Senna* spp. (Holman & Playford, 2000), and sedges (Binks, Millar, & Byrne, 2015). A similar association between clonality and aridity has been reported for animal species including grasshoppers (Kearney & Blacket, 2008) and geckos (Moritz, 2003).

Investment in asexual reproduction in harsh, relatively stable environments may offer several potential advantages over sexual reproduction (Maynard Smith, 1978; Meirmans, Meirmans, & Kirkendall, 2012; Williams, 1975). Possible explanations for the relatively high incidence of asexual reproduction in arid environments include: “a risk‐spreading mechanism that enables independent mortality of integrated hydraulic units or ramets” (Schenk, 1999); the “Ultimate Self” hypothesis which proposes that a single genotype may have evolved that is well suited to all local environmental variation (Hopper, 2009; Hopper & Barlow, 2000); or, as a mechanism through which to circumvent local or landscape‐scale environmental conditions that preclude sexual reproduction or seedling recruitment (Del Carmen Mandujano, Montaña, Méndez, & Golubov, 1998; Flores‐Torres & Montaña, 2012; Wesche, Ronnenberg, & Hensen, 2005).

Our recent study on the vulnerable canopy forming tree, *Acacia loderi*, further highlights that within the Australian arid zone, there is an unusually high frequency of asexuality, polyploidy, or both (Roberts, Forrest, Denham, & Ayre, 2016). Indeed, eight stands of *A. loderi* to the east of the Darling River were found to be genetically diverse, a result of exclusively sexual reproduction and recruitment in their semi‐arid environment. In contrast, stands further west, beyond the Darling River and its expansive floodplain and in areas considered “arid,” were highly clonal and sometimes polyploid. This variation in life history displayed by *A. loderi* follows an apparent east–west gradient of increasing aridity, and hence, the extreme clonality in more arid western sites might reflect a long history of isolation or adaptation to harsh but relatively constant conditions. A lack of genetic and genotypic diversity within western stands implies that *A. loderi*, within that region, may lack the capacity to adapt to future challenges, including impacts of disease or increased aridification of its habitat by the predicted effects of a warming climate. Nevertheless, the relatively great genetic diversity and apparent genetic interconnection of semi‐arid eastern stands implies that *A. loderi*, as a species, may be resilient despite the threat presented by habitat fragmentation and disturbance throughout much of its range (Roberts et al., 2016).

**Figure 1:**
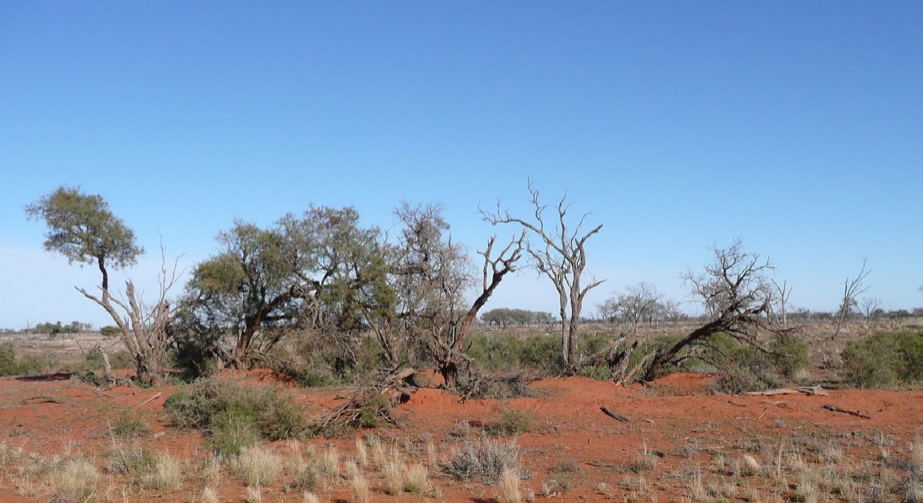
A typical stand of *Acacia carneorum* in northwest New South Wales, Australia, showing mortality of adult ramets of the same genet, probably due to rabbit warrens (visible at base) in combination with herbivory, with young suckers (asexual, vegetative growth) <1 m tall growing nearby

Within the Australian arid zone, the nationally vulnerable Purple Wood Wattle, *Acacia carneorum* Maiden, has an extensive although highly fragmented distribution, comparable in size to that of *A. loderi. Acacia carneorum* woodlands provide critical habitat for a variety of species, but in contrast to *A. loderi*, its distribution is restricted to the arid zone west of the Darling River (Auld, 1993). As for *A. loderi*, since the commencement of European pastoralism, *A. carneorum* has been exposed to elevated levels of herbivory throughout most of its range, particularly from introduced sheep, rabbits, and goats (Fig. 1). Herbivory is expected to limit the establishment of sexual recruits, that is, seedlings. *Acacia carneorum* may be more vulnerable to extinction than *A. loderi*, if it fits the general pattern of increased clonality beyond the Darling River, because stands will display low genetic diversity and little connectivity if both pollination and seed dispersal are rare.

Several lines of evidence suggest that despite the annual production of flowers, *A. carneorum* is currently almost entirely reliant on asexual reproduction for persistence in its arid habitat. First, a more than 20‐year longitudinal demographic study of stand structure and recent on‐ground surveys (Auld, T. D. & Denham, A. J., unpublished data) have found only three stands that show evidence of annual seed set, and even within these stands, few fruits per plant are produced. Furthermore, these seeds are likely to fail to germinate or grow under persistent drought conditions and in the face of extraordinarily high levels of herbivory. Second, surveys of flower visitation and inflorescence development within a small number of stands have shown that the hermaphroditic flowers receive a diverse suite of insect visitors (mainly species within the Orders Hymenoptera, Coleoptera, Lepidoptera, and Diptera). Moreover, visits per inflorescence are infrequent, and the insects carry very little pollen and hence are probably ineffective pollinators (Gilpin, Ayre, & Denham, 2014). Finally, a recent preliminary survey of genetic population structure using AFLP markers found that all surveyed stands were dominated by a small number of highly replicated multi‐locus genotypes or genets (O'Brien, Denham, & Ayre, 2014). However, O'Brien et al. (2014) surveyed only a small number of individuals (*n* = 15 per stand) within each of 10 stands using dominant AFLP markers. These markers cannot be used to detect polyploidy and their characteristically high genotyping error rate and efficiency in detecting somatic mutation may have caused an overestimation of genotypic variability.

**Figure 2:**
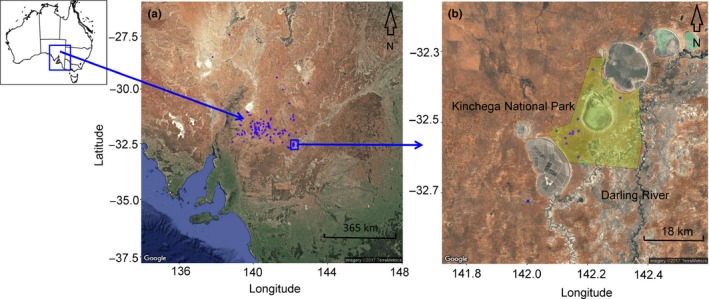
Map of the study area and the location of extant stands of *Acacia carneorum* (a). All stands were located west of the Darling River in western New South Wales. The genetic survey concentrated on stands within Kinchega National Park, western New South Wales (b). Satellite images (a) and (b) from Google Earth http://earth.google.com/

Here, we surveyed genetic variation at co‐dominant microsatellite loci in the stands surveyed by O'Brien et al. (2014) and an additional 10 *A. carneorum* stands. This allowed us to further test for geographic variation in life histories and to compare standing levels of genetic and genotypic diversity. In particular, we set out to characterize the genotypic composition of *A. carneorum* stands arrayed along the western boundary of the Darling River floodplain, the species' current eastern range limit, including stands under protection within Kinchega National Park, western New South Wales, with others found within the arid zone of eastern South Australia (Fig. 2). We consolidated herbarium records to estimate the number and location of extant stands and we further aimed to assess its conservation status by comparing genetic diversity within protected stands in National Parks and heavily grazed range lands. We use these data to expand knowledge of the distribution of genetic diversity within this species and to make recommendations for its conservation and management.

## 2 MATERIAL AND METHODS

### 2.1 Estimating the number extant stands of *Acacia carneorum*

As a vulnerable species, records of the distribution of *Acacia carneorum* feature in the databases of many state and national herbaria (Canberra, New South Wales, Melbourne, Adelaide and Western Australia). However, there has been no attempt to consolidate this information. Here, we combined existing records of occurrence with on‐ground survey data to estimate the number of extant stands of *A. carneorum* across its range. We had the additional aim of determining the number of stands that are located within existing conservation reserves. Stands vary enormously in numbers of stems, density, and area occupied. Several stands comprised single stems and others >10,000 stems (O'Brien et al., 2014). Location data from these sources were mapped to facilitate decisions regarding the likelihood of duplicate records from the same stand or herbarium collection. We devised decision rules and applied them during this process. Each record was considered and retained if: (1) The location was adequately described and was sufficiently precise to distinguish it from others in the vicinity; (2) the location is likely to have suitable habitat for the plant (e.g., the town of Broken Hill is not likely to have suitable habitat, although much occurs nearby); and (3) there was not another record already retained for that location. In general, locations needed to be separated by more than 300 m to be considered different. However, the size of stands (area of occupancy and number of stems) was estimated within several records, allowing more precise delineation of stands in some areas.

### 2.2 Genetic analyses: interpretation and analysis of microsatellite data, and genetic population structure

We generated microsatellite genotype data for a total of 20 “stands” of *A. carneorum* spread throughout its range, which is limited to western New South Wales and eastern South Australia (O'Brien et al., 2014). We re‐sampled all stems assessed by O'Brien et al. (2014) and extended our sampling to an additional 10 stands within Kinchega National Park in western New South Wales, including two stands that Auld (1993) and 20 years of subsequent demographic surveys have shown consistently produce fruits containing viable seeds (Denham, personal observation). In all stands, sampling for DNA extraction and subsequent genetic analysis involved collecting phyllodes from between eight and 52 stems per stand. We deliberately targeted large adult stems >3 m tall, separated by at least 10 m (and in some cases up to >100 m), and not clusters of putatively asexually derived root suckers (see Discussion below) to reduce the probability of sampling identical genotypes. Our sampling aimed to capture as much as possible the available genotypic diversity within each stand and sample sizes therefore varied with the absolute number of stems available for sampling in each stand. Sample sizes <30 comprised all of the available adult stems in a stand. Global positioning system coordinates were recorded for all of the sampled stems. Primers were chosen after screening 96 pairs of loci and we generated eight locus microsatellite data for all 484 sampled stems. DNA extraction, amplification of the primer set comprising eight loci, and genotype scoring followed standard laboratory protocols, the details of which are described in our earlier primer note (Roberts, Forrest, Denham, & Ayre, 2013). Collections of *A. carneorum* were approved by New South Wales Office of Environment and Heritage, under license S13104.

In scoring the microsatellite electropherograms of all 484 sampled stems of *A. carneorum,* it became apparent that the species as a whole is highly dependent on asexual reproduction. In addition, in some stands, electropherograms for one or two loci per stem presented with three visible peaks, which contrasts with the single or double peaks, respectively, for diploid homozygotes and heterozygotes. These triple peaks were repeatable using independent extractions, and their presence implies either locus duplication, for at least two of eight loci, or a polyploid genetic system with an unknown number of homologous chromosomes. Chromosome counts or other analyses to confirm ploidy unambiguously were not possible at the time of data collection, but would be informative in any future investigation.

Clonality and variation in ploidy inevitably limited the range of analyses that could be conducted. Nevertheless, this made little difference to our capacity to interpret the data set because, with the exception of two stands (that each had two putative genetic individuals), each stand appears to comprise a single ancestral genetic identity (refer to Results). We acknowledge that the 20 stands for which we had collected microsatellite data may all have a polyploid genetic system, with the clear lack of sexual recombination meaning that, in some stands, a third or fourth allele is masked, thus the presence of stands which appear apparently diploid. Indeed, in scoring the electropherograms some individuals within apparently diploid stands appeared to display dosage variation, for one of the alternative alleles. However, it was not possible to optimize the assay conditions to unambiguously score this variation. Alternatively, there may be stands of *A. carneorum* that indeed exhibit polyploidy, whereas others may be diploid. Our inability to distinguish these states meant that, in performing our analyses, we have initially been conservative, providing simple descriptive assessment of levels of genetic diversity, for each stand, and across all 20 stands surveyed. Specifically, rather than scoring each of the *n* = 484 stems as genotypes (as would be done for a true diploid), we treated the allelic composition at each locus, and summed over all loci, as a single‐ and multi‐locus phenotype (MLPs), respectively. Indeed, when a genotype cannot be determined unambiguously, then it should be treated as a phenotype rather than a genotype. Based on the MLPs for each stand, and for the entire sample of *A. carneorum*, we calculated the total number of different alleles summed across all eight loci (*A*), the mean (±SE) number of different alleles per locus (*A'*), the mean (±SE) proportion of individuals that were apparently heterozygous (*H*_o_), and the mean (±SE) observed number of alleles per individual (*A'*_MAX_). The number of apparently different MLPs was used as an estimate of the number of distinct genetic individuals within each stand, and overall, for *A. carneorum*, as a species. MLPs were found using the “matches” function implemented in genalex 6.41 (Peakall & Smouse, 2006).

**Figure 3:**
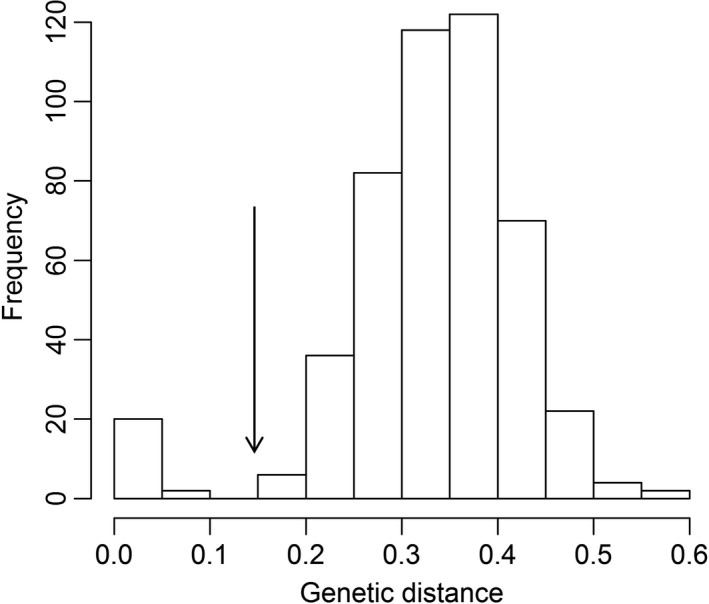
Frequency distribution of Bruvo genetic distance estimated using eight microsatellite loci, for 484 sampled stems of *Acacia carneorum* from 20 stands across the distributional range in arid western New South Wales and South Australia. A Bruvo genetic distance of 0.15 was used as the threshold to distinguish different genets (arrow)

Genotypic variation in clonal species can reflect both variation among different sexually generated clones or variants within a single genetic lineage that have arisen through somatic mutation and are expected to share almost all of the same alleles as their clonal ancestor. To distinguish between these possibilities, we used the approach of Bruvo, Michiels, D'Souza, and Schulenburg (2004) to estimate the relative genetic distance among putative diploid and polyploid individuals (Bruvo genetic distance). We used the R package polysat (tools for polyploid microsatellite analysis; Clark & Jasieniuk, 2011) to calculate Bruvo genetic distances among all stems. With two exceptions (refer below), we found that variation in MLPs (above) within stands reflected very minor variants on the numerically dominant MLP (Table 1, *N'*_MLP_). Such variation is a predictable consequence of somatic mutation (especially in long‐lived species such as perennial trees) or genotyping errors. Repeated extraction and re‐analysis minimized the possibility of genotyping errors. Bruvo genetic distance estimates for pairs of sexually produced genets (i.e., comparisons among different stands) should be much higher than for neighboring stems within the same stand, that is, ramets with a likely asexual origin. Indeed, a frequency distribution plot of Bruvo genetic distances matched this expectation, as it was clearly bimodal, with a minor peak at the lower end of the range of genetic distances (0–0.05 Bruvo genetic distances; Fig. 3). This minor peak indicates very slight genetic dissimilarity among a small number of individuals (i.e., minor variation within a single genetic lineage). To estimate the number of different genetic individuals (i.e., genets), we followed the common approach of using the genetic distance distribution to decide upon a logical genetic distance “cutoff” point which separates putatively distinct genets. Here, we used a Bruvo genetic distance of 0.15 as a threshold to distinguish different genets.

**Table 1:** Location coordinates, number of stems genotyped at eight microsatellite loci (*N*), total number of alleles across all loci (*A*), mean observed number of alleles per locus, mean (±SE) maximum number of alleles observed per individual per locus with bold values indicating apparent polyploidy (*A'*_MAX_), mean (±SE) proportion of observed heterozygotes per locus (*H*_o_), mean (±SE) observed number of unique phenotypes per locus (*N'*_SLP_), total number of observed multi‐locus phenotypes (frequency of the most common MLP; *N'*_MLP_), and total number of distinct genetic individuals (*N*_*g)*_ based on Bruvo distance matrix (threshold = 0.15), for each of 20 remnant stands of *Acacia carneorum*, southeastern Australia

Stand	Latitude	Longitude	*N*	*A*	*A'*	*A'*_MAX_	*H*_o_	*N'*_SLP_	*N'*_MLP_	*N*_*g*_
Kinchega National Park, New South Wales (NSW)										
ACAR 1	32°24.5′S	142°19.0′E	30	11	1.4 (0.2)	2 (0)	0.38 (0.18)	1.1 (0.1)	2 (0.97)	1
ACAR 2	32°32.5′S	142°9.5′E	52	13	1.6 (0.2)	2 (0)	0.62 (0.18)	1.1 (0.1)	2 (0.92)	1
ACAR 3	32°21.5′S	142°13.5′E	30	13	1.6 (0.3)	**2.1 (0.1)**	0.37 (0.18)	1.1 (0.1)	2 (0.97)	1
ACAR 4	32°33.0′S	142°7. 00′E	46	15	1.9 (0.2)	2 (0)	0.75 (0.16)	1.3 (0.2)	3 (0.96)	1
ACAR 5	32°34.0′S	142°8.0′E	31	18	2.1 (0.3)	2 (0)	0.50 (0.20)	1.9 (0.2)	4 (0.87)	2
ACAR 6	32°29.0′S	142°10.5′E	30	11	1.4 (0.2)	2 (0)	0.25 (0.16)	1.0 (0)	1 (1)	1
ACAR 7	32°31.5′S	142°9.5′E	21	13	1.6 (0.2)	2 (0)	0.50 (0.20)	1.0 (0)	1 (1)	1
ACAR 8	32°32.0′S	142°9.5′E	8	11	1.4 (0.2)	2 (0)	0.40 (0.20)	1.0 (0)	1 (1)	1
ACAR 9	32°31.5′S	142°11.0′E	31	15	1.9 (0.3)	**2.1 (0.1)**	0.63 (0.18)	1.1 (0.1)	2 (0.84)	1
ACAR 10	32°36′S	142°10.0′E	14	17	2.1 (0.1)	**2.1 (0.1)**	0.58 (0.09)	2.0 (0)	3 (0.57)	2
ACAR 14	32°31.5′S	142°9.5′E	25	15	2 (0.3)	**2.1 (0.1)**	0.74 (0.16)	1.6 (0.3)	4 (0.72)	1
Stands located West, North, and North West of Kinchega National Park, NSW										
ACAR 12	32°43.5′S	141°59.0′E	25	12	1.5 (0.2)	2 (0)	0.50 (0.19)	1.1 (0.1)	2 (0.96)	1
ACAR 11	32°9.5′S	141°56.5′E	25	16	2.0 (0.3)	2 (0)	0.72 (0.16)	1.4 (0.2)	2 (0.72)	1
ACAR 13	29°28.5′S	141°16.5′E	21	14	1.8 (0.3)	**2.1 (0.1)**	0.55 (0.18)	1.1 (0.1)	2 (0.57)	1
ACAR 15	31°25.0′S	142°11.5′E	16	13	1.6 (0.3)	2 (0)	0.49 (0.19)	1.3 (0.2)	3 (0.69)	1
ACAR 16	32°27.0′S	141°33.5′E	15	15	1.9 (0.5)	**2.25 (0.2)**	0.38 (0.18)	1.4 (0.4)	4 (0.81)	1
ACAR 17	29°44.0′S	142°58.0′E	16	16	2.0 (0.4)	**2.25 (0.2)**	0.49 (0.18)	1.4 (0.2)	5 (0.75)	1
South Australia										
ACAR 18	32°06.5′S	140°13.5′E	16	12	1.5 (0.2)	2 (0)	0.50 (0.19)	1.0 (0)	1 (1)	1
ACAR 19	32°06.5′S	139°9.0′E	16	12	1.5 (0.3)	2 (0)	0.39 (0.17)	1.3 (0.2)	3 (0.75)	1
ACAR 20	31°35.5′S	140°47.5′E	16	14	1.8 (0.3)	**2.25 (0.2)**	0.50 (0.19)	1.1 (0.1)	2 (0.94)	1
Total, species			484	44	5.5 (0.4)	**2.75 (0.2)**	0.54 (0.06)	9.5 (0.7)	49	22

Making the assumption that all of our apparently diploid genotypes are true diploids, we used probability of identity (pID), and probability of identity for siblings (pIDsib), to assess the statistical power of our marker set of eight loci to differentiate among sexually generated lineages. Using a reduced data set, that is, just 12 distinct apparently diploid genets (as revealed using a Bruvo genetic distance of 0.15), we used Genalex 6.41 to calculate the average pID that, two unrelated individuals, drawn from the same randomly mating population, will by chance have the same multilocus genotype, and the related measure pIDsib, which takes into account the genetic similarity among siblings which could clearly be relevant in small isolated stands.

## 3 RESULTS

### 3.1 The number of extant stands of *Acacia carneorum*

We obtained a total of 523 records of occurrence of *A. carneorum* from which we estimate there are 222 distinct stands, of which three are extinct.

At the time of analysis, the overall set of records represented 206 and 317 records from the New South Wales Wildlife Atlas and the Atlas of Living Australia, respectively. However, many of these records were duplicates generated by submissions from different herbaria or through repeated entry of sightings. In most cases, with the aid of collector and location descriptions, we were able to distinguish the duplicates from novel collections. Recent on‐ground survey work allowed improved precision for many previously known locations, particularly in South Australia (Playfair and McDonald personal communication). For example, three stands that are present within the records have no live plants remaining (Denham personal observation). Of the 219 putatively extant stands, 101 occur in New South Wales, including 47 from within Kinchega National Park, the only New South Wales reserve where this species has been recorded. The remaining stands are located in South Australia, where 27 are within reserves including Bimbowrie Conservation Reserve (Government of South Australian) and Boolcoomatta Bush Heritage Reserve (a private conservation reserve) and one each within the Flinders Ranges National Park and Lake Eyre National Park.

### 3.2 Genetic diversity and structure

The sample of eight microsatellite loci had ample power to distinguish between different sexually generated lineages (assuming diploidy), and each stand was genetically distinct. The estimated pID was 3.4E‐06, and pIDsib was 0.0043. Our survey of genetic diversity in 484 stems spread across twenty stands of *Acacia carneorum* revealed the overall collection of stems was genetically diverse, with the mean number of alleles per locus = 5.5 ± 0.4 and the overall proportion of observed heterozygotes per locus = 0.54 ± 0.06. However, further analysis revealed only 22 clearly independent genets, typically indicating the presence of one highly replicated genet per stand. Indeed, with only two exceptions (stand 5 and stand 10), it is probable that each stand is derived from a single ancestral genet (Table 1).

With these two exceptions, the most frequently occurring MLP in each stand was much more genetically distinct than the extremely rare minor variants detected at low frequency (<0.05) within stands, including both dominant and rare MLPs found in closely neighboring stands. Pairwise distribution of Bruvo genetic distances revealed Bruvo distance values were always >0.15 for pairs of MLPs between different stands, whereas values within stands were always <0.1. The exceptions were stand 5 and stand 10, within which Bruvo genetic distances in each case were 0.26, implying that two clonal lineages were present (Table 1). Variation between pairs of MLPs separated by Bruvo distance of ≤0.1 reflected no more than a two base pair difference in the size of one of the alleles at a single locus, that is, either a decrease or increase in allele size (base pairs) coincident with a single microsatellite repeat motif from the phenotype of the most frequently encountered genet in their stand. These rare variants were consistent with the outcome of somatic mutation of the ancestral clone rather than self‐fertilization of the dominant clone or outcrossing with other clones. Indeed, the great majority of all stands displayed fixed heterozygosity at 3–6 loci.

Genetic diversity within individual stands was low, with the average number of alleles per locus per stand ranging from 1.4 to 2.1. Despite the presence of only one or two MLPs observed per stand, heterozygosity was moderate to high, with the proportion of observed heterozygotes per locus per stand ranging from 0.25 to 0.75 (Table 1), and all phenotypes were heterozygous for three to six of the eight loci scored.

Eight of the twenty sampled stands, displayed three clear allelic peaks (indicated in bold in Table 1) as would be expected for polyploid genotypes. Stands with putatively polyploid genotypes were distributed throughout the sampled range and included four stands within Kinchega National Park (Table 1).

The extremely clonal nature of the 20 widely distributed stands of *A. carneorum* that we surveyed (each representing only one or two different genets) means that in effect our data set comprise only 22 individuals, thus precluding most standard population genetic analyses.

## 4 DISCUSSION

Our range‐wide survey of genotypic diversity in *Acacia carneorum* supports other accounts of clonal population structures in plant and animal taxa from arid environments, and the occurrence of polyploidy (Andrew et al., 2003; Kearney, 2003 and references therein; Roberts et al., 2016). Our findings of almost exclusively asexual reproduction and apparent variation in ploidy in *A. carneorum* stand in contrast to the initial finding of O'Brien et al. (2014) of moderate clonality, with multiple genotypes detected within a smaller number of samples and surveyed stands. We detected no evidence of recent connectivity through either pollen or seed dispersal, and all 20 stands surveyed displayed little genotypic diversity. In fact, our study revealed that the majority of surveyed stands comprise only one (often a putative polyploid) genetic individual. Selection within an arid and highly grazed environment may favor the persistence of locally adapted genotypes (but see review by Leimu & Fischer, 2008) although it is unclear how long such clonal lineages can persist without episodic input of genetic diversity through sexual reproduction. Nevertheless, each genet is unique, and all display high heterozygosity, a phenomenon commonly found in plant and animal clonal allolopolyploids formed through interspecific hybridization (Levin, 1983; Stebbins, 1984; Vrijenhoek, 1979). While the exact mechanism through which apparent polyploidization has arisen in *A. carneorum* is unknown, our findings are consistent with the hypothesis that, when in combination with asexual reproduction, polyploidization may facilitate habitation in extreme environments (De Witte & Stöcklin, 2010; Kearney, 2003; Levin, 1983; Stebbins, 1984; Vrijenhoek, 1979).

Our study on *A. carneorum* contrasts with earlier work on other Australian *Acacia* species for which clonality, polyploidy, or both have been documented because we detected no evidence of sexual reproduction via either cross‐ or self‐fertilization. For example, within the widespread *Acacia aneura* species complex, predominate asexual reproduction through apomixis in some stands may be an ancient condition, allowing especially well adapted genets to be long‐lived, where in other areas sexual reproduction predominates and provides an ongoing source of genetic variation (Andrew et al., 2003; Miller, Andrew, & Maslin, 2002). Several other arid zone *Acacia* species, for example, *A. homalophylla*,* A. loderi*,* A. melvillei,* and *A. pendula* (Forrest, Roberts, Denham, & Ayre, 2015), still apparently use sexual reproduction to generate seed, with genets also proliferating via the spread of ramets produced by vegetative suckering. In these species, suckering might be a response to elevated levels of disturbance, following the introduction of agricultural activities such as grazing and cultivation (Auld, 1995; Batty & Parsons, 1992; Chesterfield & Parsons, 1985). Indeed, genotypes that thrive under current conditions and which are able to readily proliferate through suckering yet invest little energy in maturation of sexual progeny (i.e., viable seed) might be strongly favored by selection. Nevertheless, because all of these species have the capacity for episodic sexual reproduction (Forrest, C. N., Denham, A. J., Roberts, D. G., & Ayre, D. J., unpublished data), the switch to greater reliance on asexual reproduction for persistence may be of recent origin. In the case of *A. carneorum*, while disturbance via herbivory may increase rates of suckering, our finding that nearly all stands, including those with 100s to 1000s of widely spaced stems, together with the widespread failure of seed set, implies that the existing stand structure might be ancient. Indeed, many stands of this species may have lost the capacity for sexual reproduction (perhaps coincident with polyploidization or increasing aridification of Australia) and thus have been dependent on asexual reproduction prior to the expansion of European agriculture. Radiocarbon dating of stems to accurately determine their ages may provide novel insights, including whether genet ages predate the onset of pastoralism (or indeed European colonization of Australia), and thus, whether the almost complete reliance on asexual reproduction is an ancient or recent phenomenon.

In contrast to the earlier work of O'Brien et al. (2014), which suggested the presence of multiple distinct genetic individuals in 8 of 10 stands, we found that despite the larger sample size within each *A. carneorum* stand that we surveyed, each stand appeared to contain either just one or, in two cases, two genets, with eight stands showing electropherograms indicative of polyploidy. While the use of dominant AFLP markers by O'Brien et al. (2014) would have prevented detection of polyploidy, their contrasting estimates of clonal diversity might reflect the higher genotyping error rates associated with the use of AFLPs (Bonin et al., 2004), or a greater capacity to detect accumulated somatic mutations (Kuchma, Vornam, & Finkeldey, 2011). With our microsatellite data set, we were able to detect evidence of polyploidy (albeit preliminary) through the presence of three peaks on electropherograms. Moreover, although genotypic variation was rare within stands, our knowledge of the microsatellite repeat motif of each locus from marker development (Roberts et al., 2013) was informative, as it allowed determination of whether slight genetic dissimilarity (as measured by base‐pair size) among sampled stems was indicative of either a decrease or increase in the size of a single repeat motif. This allowed identification of multilocus lineages, that is, slightly distinct copies (because of either genotyping error or detectable somatic mutation) of essentially the same genotype (Arnaud‐Haond, Duarte, Alberto, & Serrão, 2007). Although much remains to be learnt about the genetic system of *A. carneorum*, including unambiguous confirmation of polyploidy, our study together with the earlier work of O'Brien et al. (2014), implies that the species as a whole, is (and has been in the past) almost exclusively dependent on asexual reproduction for persistence, with virtually no genotypic diversity contained within individual stands.

Our recent study of arid, and semi‐arid Australian *A. loderi* (Roberts et al., 2016), as well as earlier studies on Mulga, *A. aneura* (Andrew et al., 2003; Miller et al., 2002), suggest a complex pattern of spatial or temporal variation with respect to the frequency of sexual reproduction, levels of ploidy (which for Mulga may be associated with hybridization), and methods of asexual reproduction (suckering cf. apomictic seed production). For example, in *Acacia loderi*, stands that are almost entirely sexually derived were found in semi‐arid parts of its range to the east of the Darling River, whereas some of its western stands are strongly reliant on asexual reproduction through root suckering and display little genetic diversity (Roberts et al., 2016). *Acacia carneorum*, like these western stands of *A. loderi*, appears to consist of stands with varying levels of ploidy and capacity for sexual generation of seed. However, without intervention, the severe fragmentation of this species and the monoclonal nature of most stands suggest that it will lose all natural capacity to generate new genotypic diversity through outcross fertilization and recombination.

It is unclear for *A. carneorum* how much genetic and genotypic diversity has been lost through fragmentation for farming and reduction in area of occupancy or the effect on resilience of this loss of diversity. Even if this species was highly clonal prior to the expansion of pastoralism in the mid‐1800s (Caughley, 1987), our data still imply that considerable numbers of potentially distinct and ancient genets have been lost because the species as a whole retains moderate genetic variation (mean = 5.5 alleles/locus), even though most stands contain just one genotype. However, genets may persist or be long‐lived because they represent good “general purpose genotypes” (Baker, 1965; Parker, Rodriguez, & Loik, 2003; Scott, Meyer, Merrill, & Anderson, 2010) or because they represent the “ultimate self” (Hopper, 2009; Hopper & Barlow, 2000). Alternatively, extant clonal diversity could be an example of the diversity associated with the phenomena of recently “lost sex” (Vrijenhoek & Parker, 2009; Wilson & Hebert, 1992), comprising a small and potentially random subset of the genotypic diversity generated by the most recent episode of sexual recruitment (perhaps coincident with pastoral expansion and natural selection favoring asexual reproduction; McAlpine et al., 2009). Whatever the origin of the existing genotypes, it appears likely that fragmentation mediated by the expansion of pastoralism has both reduced local genotypic diversity and the probability that compatible mating partners occur in sufficiently close proximity to allow outcross pollination and subsequent sexual reproduction.

Taken together our genetic data set and analysis of records of occurrence imply that *A. carneorum* as a species may now comprise relatively few genetic individuals with most being isolated from other potential mates by tens or hundreds of kilometers. In addition to the surprising lack of genotypic diversity present with the surveyed stands of *A. carneorum*, our analysis of herbarium records for *A. carneorum* also provides a bleak picture. While some stands of *A. carneorum* comprise a thousand or more individual stems, these are the exception (O'Brien et al., 2014), with most stands comprising far fewer, and in some cases lone stems. Importantly, the number of apparently separate stands recorded within all available databases greatly overestimates the real number of extant stands; from over 500 records we estimate that 219 extant stands of *A. carneorum* remain. Our range‐wide genetic analysis of 20 implies that on average, each of the stands represents 1.1 genetic individuals, suggesting an overall population of about 240 genetically distinct individuals. While our data revealed the presence of minor mutational variants on the core genet of a stand, these were detected relatively rarely in our microsatellite survey (although refer to discussion above regarding possible detection using AFLP) and such variation is unlikely to signify substantial variation in fitness among ramets. Indeed, somatic mutations would be unlikely to be expressed as they would typically be recessive with mutant alleles present within heterozygous genotypes (Kirkpatrick & Jenkins, 1989).

### 4.1 Implications for conservation and management

Our data imply that the conservation status of *A. carneorum* should be revised to “Endangered” rather than its current status of “Vulnerable.” The basis for this classification is criterion D of The IUCN Red List of Threatened Species (http://www.iucnredlist.org/static/categories_criteria_3_1), which indicates that “a taxon is endangered when the best available information indicates that (its) population size (is) estimated to number fewer than 250 mature individuals.” Currently, *A. carneorum* does not meet this criterion based on demographic survey data alone; some stands number 100s or up to a 1000 or more stems (O'Brien et al., 2014). The IUCN criteria include counts of ramets, except where they are unable to survive alone or will never produce new recruits. Underestimation of population size in clonal plants is therefore likely to be a general phenomenon, one which has important implications for conservation (Tepedino, 2012). Indeed in applying IUCN criterion D to *A. carneorum*, our data indicate that the species as a whole likely comprises only slightly more than 219 distinct genetic individuals (the number of extant stands), and each stand may in effect represent a single genet (or two, in two cases herein). Thus, most of the stands that we surveyed probably have effective population sizes less than two and at least without intervention appear unable to generate seed.

*Acacia carneorum* is not yet obligately asexual; it retains the capacity for episodic sexual reproduction in at least three known “fruiting” stands. However, it is a concern that even fruiting stands contain just one genet per stand, suggesting that recruitment via sexual reproduction has been persistently unsuccessful. Seeds generated within these stands are genotypically diverse, and paternity analysis has revealed genetic input from at least two neighboring stands (Forrest, C. N., Denham, A. J., Roberts, D. G. & Ayre, D. J., unpublished data.). Thus, these stands could be used to produce seedlings for ex situ conservation or be protected from herbivory in situ by protective barriers (see Auld, 1993; Denham & Auld, 2004). Thirty‐four percent of extant stands of *A. carneorum* are contained within parks or reserves and we argue that Kinchega National Park and other similar reserves should be the focus of on‐ground conservation efforts. These should include greater protection of fruiting sites (and neighboring stands that are a source of pollen) against excessive herbivory, primarily from feral herbivores (Auld, 1993; Denham & Auld, 2004), as well as attempts to augment genetic diversity in these stands.

## CONFLICT OF INTEREST

None declared.
